# Multimodal ultrasonographic and clinicopathological model for predicting high-volume lymph node metastasis in cN0 papillary thyroid carcinoma

**DOI:** 10.3389/fendo.2025.1613672

**Published:** 2025-08-21

**Authors:** Jiwen Qian, Zheng Zhang, Yanwei Chen, Shuangshuang Zhao, Wenjun Li, Jiayan Bao, Huajiao Zhao, Yun Cai, Baoding Chen

**Affiliations:** Department of Medical Ultrasound, Affiliated Hospital of Jiangsu University, Zhenjiang, China

**Keywords:** high-volume lymph node metastasis, ultrasonography, ACR scores, papillary thyroid carcinoma, CEUS

## Abstract

**Background:**

Given the challenge in preoperative diagnosis of high-volume lymph node metastasis (HVLNM) in clinical practice, we constructed and externally validated a comprehensive predictive model that integrated conventional ultrasound characteristics, contrast-enhanced ultrasound (CEUS) parameters, BRAF^V600E^mutation, and clinicopathological data for HVLNM in clinically lymph node-negative (cN0) papillary thyroid carcinoma (PTC).

**Methods:**

Totally, 126 clinically lymph node-negative (cN0) PTC patients who underwent subtotal or total thyroidectomy and accompanied with prophylactic cervical lymph node dissection between December 2022 and December 2024 were enrolled in this retrospective study, and an additional 47 cN0 PTC patients included for the external validation cohort. Univariate and multivariate analysis were performed to identify the independent risk factors for HVLNM, and a binary logistic regression equation and relevant nomogram was constructed to predict the risk about HVLNM. The model underwent internal validation using ten-fold cross-validation and further externally validated in an independent external cohort. Clinical practicality of the nomogram model was assessed by the area under the curve (AUC), calibration curve, and decision curve analysis (DCA).

**Results:**

Age, Dmax, ACR scores ≥11 points, and heterogeneous enhancement were four independent predictors of HVLNM after univariate and multivariate analysis in training set. These predictors were used to construct the corresponding nomograms with AUC of 0.860(95% CI: 0.792-0.928). Calibration curves and DCA plots revealed their robust calibration performances and fine net benefits. The accuracy and Kappa value obtained through ten-fold cross-validation were 0.864 and 0.468. The ROC value of the external validation was 0.885(95% CI:0.792-0.978).

**Conclusion:**

Our visualization nomogram provides clinicians with useful information in a simple and cost-effective manner, aiding in the formulation of personalized treatment plans and the reduction of reoperation rates.

## 1 Introduction

Papillary thyroid carcinoma (PTC) represents the most prevalent histological subtype of thyroid cancer, with lymph node metastasis (LNM) in 40-90% PTC patients according to recent global cancer statistics (1, 2), Even in clinically lymph node-negative (cN0) which was defined as no clinical or imaging evidence of node metastasis (3), pathologic lymph node metastases are found in 30-50% when prophylactic central neck dissection is performed (4, 5). LNM has been established as a significant risk factor adversely influencing the survival rate (6) especially disease-free survival (DFS) in PTC patients and is strongly related to poor prognosis (7), disease recurrence, and distant metastasis (8).

Lymph node management remains controversial while surgery remains the cornerstone of papillary thyroid carcinoma (PTC) treatment. Critical adverse features like high-volume lymph node metastasis (HVLNM) (9) which is defined as metastasis more than five lymph nodes with all involved lymph nodes measuring <3 cm in the largest dimension according to American Thyroid Association (ATA) (9) guidelines can only be confirmed postoperatively. ATA reclassified HVLNM as intermediate-risk, which requires completion thyroidectomy and leads to higher complication risks, patient distress, and healthcare costs compared to definitive initial surgery. A systematic review revealed that the number of positive lymph nodes significantly correlated with recurrence risk in patients with regional lymph node metastasis. The study demonstrated markedly different median recurrence rates among pathologic Nodal stage1(pN1) patients based on nodal burden: 4% (range 3%-8%) for those with <5 metastatic nodes versus 19% (range 7%-21%) for those with >5 metastatic nodes (10).

Ultrasound (US) is recommended as the first-line imaging modality for PTC and LNM examination due to convenient, non-invasive and low-cost capabilities (11, 12). However, its diagnostic accuracy is limited by operator dependency and suboptimal sensitivity (13, 14). Current evidence demonstrates that CT exhibits relatively low sensitivity in detecting micrometastases and morphologically normal metastatic lymph nodes, primarily due to the inherent physical constraints of its spatial resolution (15, 16). Fine-needle aspiration cytology (FNAC) is considered as another method for diagnosing both PTC and LNM, nevertheless, its clinical utility is often restricted by complex anatomy in the cervical region, particularly when target lesions are inaccessible for percutaneous sampling (17). Despite advances in diagnostic imaging, the reliable preoperative evaluation of high-volume lymph node metastasis (HVLNM) continues to pose significant clinical difficulties. Our research represents the first attempt to develop a nomogram model integrating ACR scores, clinicopathological characteristics, and contrast-enhanced ultrasound parameters for the prediction of high-volume lymph node metastasis (HVLNM) which is expected to provide clinicians to guide optimal surgical planning and reduce the reoperation rate.

## 2 Materials and methods

### 2.1 Ethical considerations

The study protocol received ethical approval from the Institutional Review Board (approval number: SWYXLL20192252). The requirement for informed consent was waived owing to the retrospective study design, and the patient’s personal information was strictly protected.

### 2.2 Patient selection

**Figure 1:**
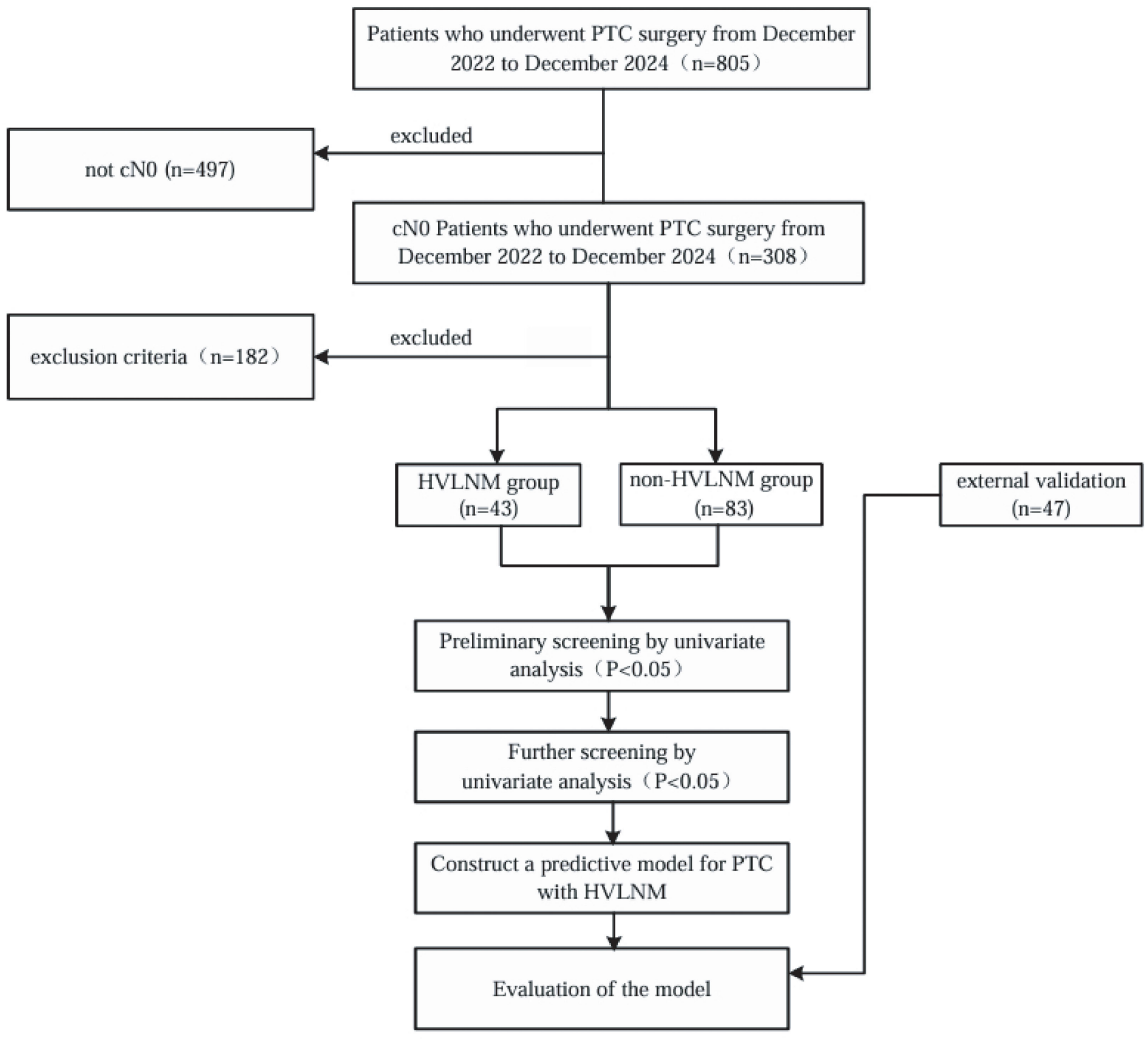
Flowchart of the study.

This retrospective study included a cohort of 126 cN0 PTC patients with 126 evaluable nodules (For each participant, the highest-risk lesion was selected for analysis.) who underwent subtotal or total thyroidectomy and accompanied with prophylactic cervical lymph node dissection between December 2022 and December 2024 at the Affiliated Hospital of Jiangsu University. Additional 47 cN0 PTC patients who received same treatment at the Zhenjiang First People’s Hospital from December 2023 to December 2024 were included as an external validation cohort. Inclusion criteria: 1) Postoperative pathology examinations confirmed diagnosis of PTC, with all patients having no suspicious lymph nodes detected on imaging examinations. Exclusion criteria: 1) Incompletion of serological tests which including a thyroid panel of seven tests and calcitonin levels, within one month prior to surgery; 2) Unavailability of US examinations, including conventional and contrast-enhanced ultrasound (CEUS) within one month before surgery; 3) Incompletion of preoperative fine-needle aspiration (FNAC) and BRAF^V600E^ gene tests; 4) Mixed-type thyroid cancer containing non-papillary components; 5) concurrent diagnosis of secondary primary malignancies, including but not limited to breast carcinoma, gastric adenocarcinoma, or follicular variant of non-Hodgkin lymphoma; 6) prior therapeutic interventions involving thyroid ablation procedures or any surgical procedures in the cervical region. The flowchart of this study is shown in **Figure 1**.

### 2.3 Clinical and biochemical information

The baseline demographic characteristics encompassed age and gender distribution (male vs. female). Comprehensive biochemical profiling included the following parameters: calcitonin (CT), free triiodothyronine (FT3), free thyroxine (FT4), thyroglobulin (Tg), thyroglobulin antibody (Tg-Ab), thyrotropin receptor antibody (TR-Ab), thyroid-stimulating hormone (TSH) and thyroid peroxidase antibody (TPO-Ab). Reference ranges for these biochemical markers were established based on clinical laboratory standards: CT (0–18 pg/mL), FT3 (3.1- 6.8 pmol/L), FT4 (12–22 pmol/L), Tg (3.5–77 ng/mL), Tg-Ab (0–115 IU/mL), TR-Ab (0-1.5U/L), TSH (0.27- 4.2 mIU/mL), and TPO-Ab (0–34 IU/mL).

### 2.4 Detection of BRAF V600E mutation

This kit (Beijing ACCB Biotech Ltd, Beijing, China) utilizes fluorescence PCR technology, employing the Amplification Refractory Mutation System (ARMS) to design specific primers. When the DNA template is of mutant type, the ARMS primers bind to the template, enabling Taq DNA polymerase to use deoxynucleotides (dNTPs) as substrates for *in vitro* amplification of the specific mutant region of the BRAF gene. The process is monitored in real-time through the release of fluorescence by hydrolysis of specific probes during the PCR reaction. Mutation analysis was conducted at Jiaxing ACCB Biotech Laboratory Co., Ltd. The PCR kit has a limit of detection (LOD) for BRAF^V600E^ as low as 1% (as per the PCR kit instructions). All results were confirmed in accordance with the standards recommended by the manufacturer.

### 2.5 Preoperative ultrasound examination

The conventional ultrasound examinations were independently performed by two certified sonographers, with more than 10 years of specialized experience in thyroid detection. Intraclass correlation coefficient (ICC) evaluated inter-observer and intra-observer reproducibility. All CEUS images were independently assessed by two board-certified sonographers, each with over 10 years of specialized experience in thyroid imaging. Cases with discordant interpretations (n=23) were adjudicated by a senior attending radiologist with more than 15 years expertise. In the training set, all conventional and CEUS examinations were performed using the Aplio i800 (Canon) equipped with 10–18 MHz linear array transducers. In addition, in the external validation set, all conventional and CEUS examinations were conducted using the Resona R9 (Mindray) equipped with 5–18 MHz linear array transducers. Digital ultrasound images were archived for subsequent quantitative analysis. For CEUS examinations, sulfur hexafluoride microbubbles (SonoVue), a pure blood pool contrast agent, was administered intravenously. The qualitative analysis of thyroid nodule enhancement patterns was restricted to CEUS recordings meeting the following criteria: 1) continuous imaging duration exceeding 1 minute; 2) the nodule fully appearing in the observed image; 3) no significant shift of the nodule.

Conventional-US parameters were included: based on multifocality, location, diffuse thyroid disease, nodule max-diameter, blood flow, capsule contact, and ACR scores. The ACR TI-RADS classification system evaluates thyroid nodules based on five key sonographic features (18): 1) Echogenicity (anechoic, 0 point; isoechoicor hyperechoic,1 point; hypoechoic,2 points; markedly hypoechoic,3 points), 2) Composition (spongiform or cystic or almost cystic,0 point; mixed cystic-solid,1 point; >95% solid, 2 points), 3) Shape (wider-than-tall,0 point; taller-than-wide, 3 points), 4) Calcification (none or comet-tailartifacts,0 points; macrocalcifications,1 point; peripheral calcifications,2 points; microcalcifications,3 points), 5) Margin (smooth or well-defined, 0 point; irregular or lobulated, 2 points; extrathyroidal extension,3 points). Draw on prior relevant research of LNM in PTC, cumulative scores were stratified into 3 categories: 5 points, 6–10 points, and ≥11 points. CEUS parameters primarily focused on (19): enhancement type (iso-or hyper-, hypo-), enhancement homogeneity (homogeneous, heterogeneous), peripheral enhancement ring (present, absent), and enhancement pattern (diffuse, centrifugal, centripetal).

### 2.6 Statistical analysis

Statistical analyses were performed by using SPSS version 27.0 (IBM) and R programming software (version 4.4.2). The Shapiro-Wilk test and Bartlett’s test were used to assess data normality and homogeneity of variance, respectively. Normally distributed continuous variables with equal variances were expressed as mean ± standard deviation (mean ± SD), while non-normally distributed variables were summarized as median (interquartile range, IQR). Categorical variables were presented as frequencies (%). For between-group comparisons, Mann-Whitney U test was utilized when the assumption of homogeneity of variance was violated. Chi-squared test or Fisher’s exact test were performed for univariate analyses of categorical variables. The discriminative performance of our predictive model was evaluated by using the AUC. Model’s calibration curves assess the agreement between predicted probabilities and observed outcomes. The clinical utility of the model was further examined through decision curve analysis (DCA), which quantifies the net benefit across a range of threshold probabilities. Internal validation was conducted using ten-fold cross-validation, with model performance evaluated based on accuracy and the kappa coefficient for inter-rater agreement. Statistical significance was set at P-value < 0.05.

## 3 Results

### 3.1 Baseline characteristics and univariate analysis

This retrospective study enrolled 126 consecutive patients (mean age: 47.0 ± 12.2 years) after employing propensity score matching (PSM) to control for confounding variables (see details in **Supplementary Table 1**), stratified into two cohorts based on LNM status by postoperative pathology: the HVLNM group (n=43) and the non-HVLNM group (n=83). Genetic analysis identified BRAF^V600E^ mutations in 115 cases (89.8%). Univariate analysis revealed six significant intergroup differences as followed: age (P=0.01), Tg (P=0.008), max nodule diameter (Dmax) (P<0.001), ACR scores (P=0.013), capsular contact (P<0.001), and homogeneity(P<0.001) (**Tables 1**–**3**). Inter-rater reliability analysis demonstrated excellent intra-observer agreement for both sonographers, with ICC ranging from 0.780 to 0.946 and 0.769 to 0.926, respectively. The inter-observer agreement between two sonographers was also substantial, with ICC ranging from 0.773 to 0.928 (**Table 4**).

**Table 1:** Baseline data comparison in HVLNM and non-HVLNM cohort of training and external test set.

Variables	HVLNM train set (N=43)	non-HVLNM train set (N=83)	*P*	HVLNM external set (N=15)	non-HVLNM external set (N=32)	*P*
Age	38 (34~48)	47 (38~57.25)	**0.01**	35 (24~45)	47.5 (39~57.75)	**0.004**
Gender			0.60			0.40
male	16 (37.2)	27 (32.5)		10 (66.7)	25 (78.1)	
female	27 (62.8)	56 (67.5)		5 (33.3)	7 (21.9)	
FT3 (pmol/L)	5.06 (4.48~5.93)	5.23 (4.76~5.60)	0.78	4.95 (4.42~5.6)	5.22 (4.95~5.62)	0.37
FT4 (pmol/L)	17.37 (16.06~19.41)	17.38 (15.86~19.17)	0.62	17.52 (15.75~20.26)	17.90 (16.53~20.62)	0.72
TSH (uIU/ml)	1.73 (1.08~2.62)	2.035 (1.35~2.84)	0.17	1.77 (1.23~2.92)	1.86 (1.35~2.62)	0.59
Tg-Ab (IU/ml)	17.26 (15.22~91.38)	18.03 (13.99~42.82)	0.60	16.39 (15.38~54.61)	19.97 (17.40~68.66)	0.05
TPO-Ab (IU/ml)	2.61 (1.44~8.15)	2.8 (0.96~37.18)	0.62	5.39 (1.84~140.2)	3.99 (1.25~50.38)	0.72
Tg (ng/ml)	30.7 (7.4~68.8)	14.1 (6.3~27.75)	**0.008**	21.2 (3.7~52.1)	10.8 (6.98~26.33)	0.56
TR-Ab (U/L)	0.25 (0.25~0.46)	0.335 (0.25~0.53)	0.09	0.3 (0.25~0.45)	0.36 (0.26~0.55)	0.14
CT (pg/ml)	3.04 (2~6.11)	3.46 (2~8.89)	0.78	3.27 (2~10.2)	3.08 (2~9.03)	0.78
BRAF gene			0.38			0.39
positive	40 (93)	73 (88)		14 (93.3)	27 (84.4)	
negative	3 (7)	10 (12)		1 (6.7)	5 (15.6)	

Non-normally distributed variables were summarized as median (interquartile range, IQR). Categorical variables were presented as frequencies (%). *P*, P value.

FT3, free triiodothyronine; FT4, free thyroxine; TSH, thyroid-stimulating hormone; Tg-Ab, thyroglobulin antibody; TPO-Ab, thyroid peroxidase antibody; Tg, thyroglobulin; TR-Ab, thyrotropin receptor antibody; CT, calcitonin.

Bold values denote statistically significant differences (p < 0.05).

**Table 2:** Conventional US comparison in HVLNM and non-HVLNM cohort of training and external test set.

Variables	HVLNM train set (N=43)	non-HVLNM train set (N=83)	*P*	HVLNM external set (N=15)	non-HVLNM external set (N=32)	*P*
Diffuse lesions			0.68			0.99
present	7 (16.3)	16 (19.3)		7 (46.7)	15 (46.9)	
absent	36 (83.7)	67 (80.7)		8 (53.3)	17 (53.1)	
Dmax			**<0.001**			**0.02**
<10mm	9 (18.8)	59 (71.1)		3 (20)	18 (56.3)	
≥10mm	39 (81.3)	24 (28.9)		12 (80)	14 (43.8)	
Multi-focality			0.69			0.78
present	30 (69.8)	55 (66.3)		10 (66.7)	20 (62.5)	
absent	13 (30.2)	28 (33.7)		5 (33.3)	12 (37.5)	
Tumor position			0.36			0.99
left	15 (34.9)	40 (48.2)		6 (40)	14 (41.2)	
right	24 (55.8)	37 (44.6)		7 (46.7)	16 (47.1)	
isthmus	4 (9.3)	6 (7.2)		2 (13.3)	4 (11.8)	
Capsule contact			**<0.001**			**<0.001**
present	26 (60.5)	21 (25.3)		11 (73.3)	7 (21.9)	
absent	17 (39.5)	62 (74.7)		4 (26.7)	25 (78.1)	
Blood flow			0.36			0.87
no	8 (18.6)	28 (33.7)		5 (33.3)	7 (21.9)	
slight	27 (62.8)	43 (51.8)		7 (46.7)	17 (51.3)	
middle	6 (14.0)	9 (10.8)		2 (13.3)	5 (15.6)	
rich	2 (4.7)	3 (3.6)		1 (6.7)	3 (9.4)	
ACR scores			**0.01**			**<0.001**
≤5	1 (2.3)	10 (12)		0 (0)	4 (12.5)	
6-10	28 (65.1)	62 (74.7)		2 (13.3)	23 (71.9)	
≥11	14 (32.6)	11 (13.3)		13 (86.7)	5 (15.6)	

Data are percentages, with numerators/denominators in parentheses.

Bold values denote statistically significant differences (p < 0.05).

**Table 3:** CEUS comparison in HVLNM and non-HVLNM cohort of training and external test set.

Variables	HVLNM train set (N=43)	non-HVLNM train set (N=83)	*P*	HVLNM external set (N=15)	HVLNM external set (N=32)	*P*
Enhancement type			0.52			0.39
hyper- or iso- enhancement	39 (90.7)	72 (86.7)		14 (93.3)	27 (84.4)	
hypo enhancement	4 (9.3)	11 (13.3)		1 (6.7)	5 (15.6)	
Homogeneity			**<0.001**			0.05
heterogeneous	22 (51.2)	18 (21.7)		9 (60)	6 (18.8)	
homogeneous	21 (44.8)	65 (78.3)		6 (40)	26 (81.3)	
Surrounding enhancement ring			0.93			0.71
present	35 (81.4)	67 (80.7)		12 (80)	24 (75)	
absent	8 (18.6)	16 (19.3)		3 (20)	8 (25)	
Perfusion pattern			0.90			0.84
centripetal	2 (4.7)	3 (3.6)		1 (6.7)	2 (6.3)	
centrifugal	19 (44.2)	34 (41)		3 (73.3)	9 (28.1)	
Diffuse	22 (51.2)	46 (55.4)		11 (20.0)	21 (65.6)	

Bold values denote statistically significant differences (p < 0.05).

**Table 4:** ICC of Intra- and Inter- observer agreements in conventional US.

Kappa coefficient	Intra-observer agreement		Inter-observer agreement	P-value
	(n=126)	(n=126)	(n=126)	
Diffuse thyroid disease	0.860 ± 0.055	0.792 ± 0.066	0.807 ± 0.065	<0.01
ACR scores	0.902 ± 0.043	0.863 ± 0.050	0.900 ± 0.044	<0.01
Capsule contact	0.886 ± 0.042	0.917 ± 0.036	0.869 ± 0.045	<0.01
Blood flow	0.855 ± 0.042	0.869 ± 0.040	0.814 ± 0.048	<0.01
Dmax	0.855 ± 0.057	0.851 ± 0.059	0.851 ± 0.059	<0.01

### 3.2 Multivariate analysis

Six variables with statistical significance showing after univariate analysis were then conducted in multivariate logistic regression model. Four independent factors of age (OR=0.959, 95% CI:[0.924-0.995], P=0.024), Dmax ≥10mm (OR=12.544, 95% CI:[4.488-35.06], P<0.001), ACR scores ≥ 11 points (OR=34.986,95% CI: [2.208 -492.781], P=0.011), and heterogeneous enhancement (OR=4.076, 95% CI: [1.469 -11.308], P=0.007) were identified for predicting high-volume lymph node metastasis (HVLNM) (**Table 5**).

**Table 5:** Multivariate analysis in training cohort.

Variables	β	S.E.	W	OR (95% CI)	P-value
Age	-0.042	0.019	5.074	0.96 (0.92~0.99)	0.024
Dmax	2.529	0.524	22.263	12.54 (4.49~35.06)	<0.001
ACR scores	3.496	1.38	6.422	32.98 (2.21~592.78)	0.011
Homogeneity	1.405	0.521	7.285	4.076 (1.47~11.31)	0.007

β, regression coefficient; S.E., Standard Error; W, Wald; OR, Odds Ratio; CI, confident interval.

### 3.3 Construction of the model

**Figure 2:**
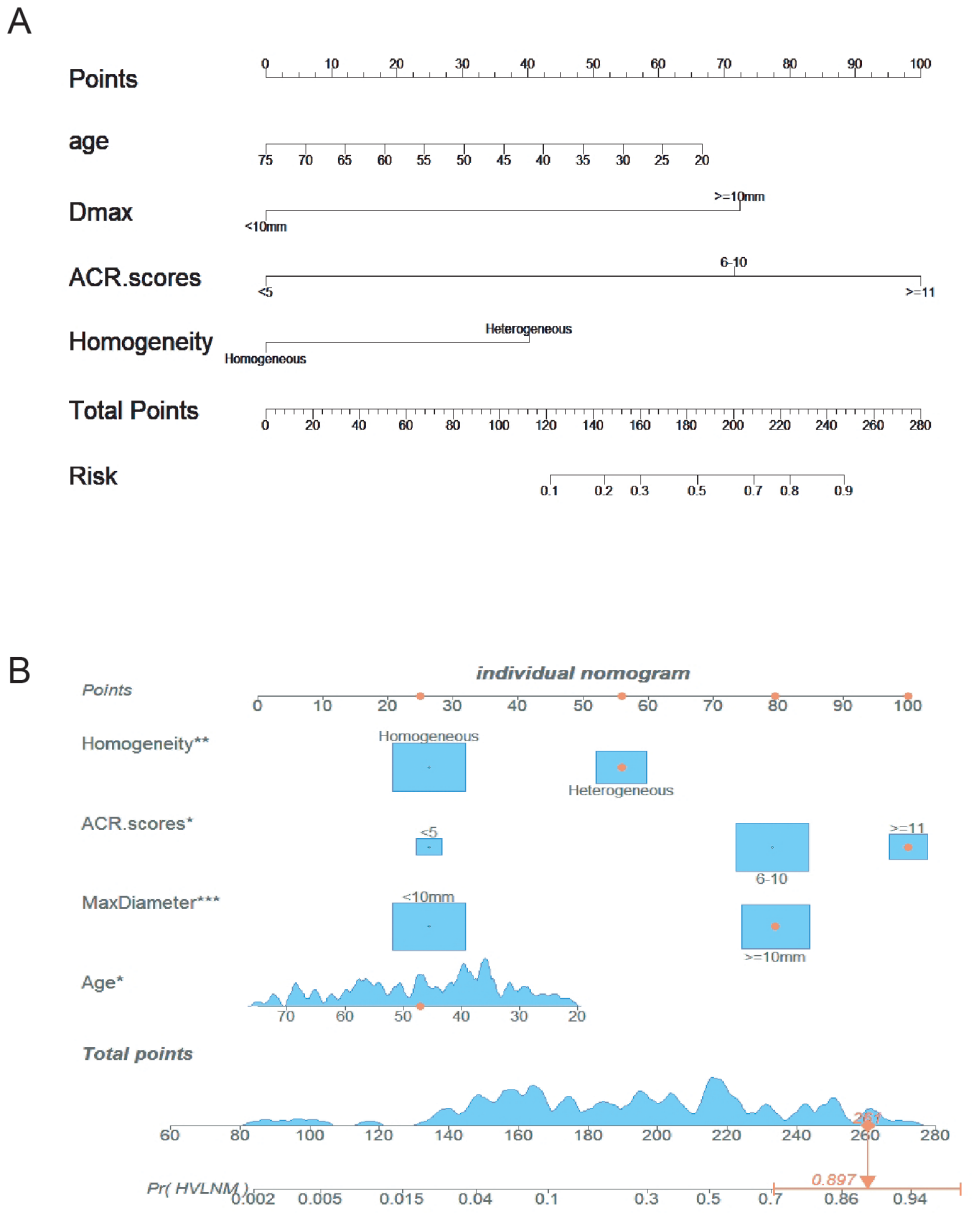
**(A)** Nomogram for predicting HVLNM in PTC patients. **(B)** Add a case to the nomogram for predicting HVLNM in PTC patients. * means p < 0.05, ** means p < 0.01,*** means p < 0.001.

**Figure 3:**
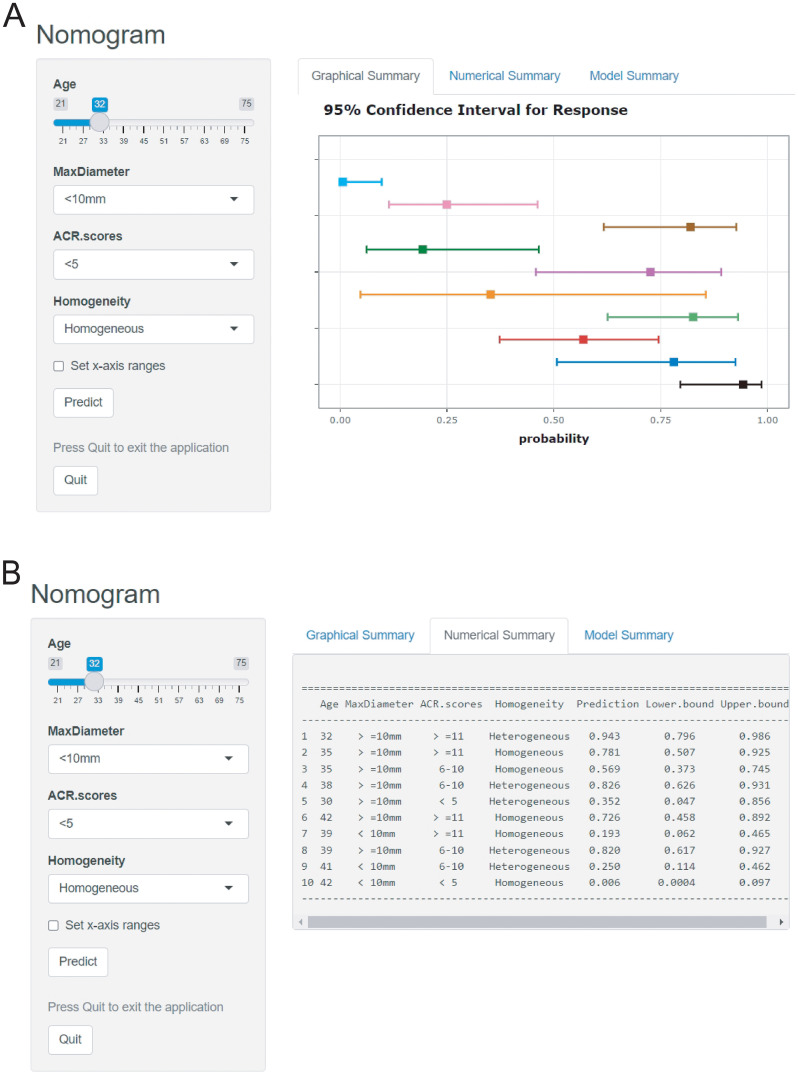
**(A, B)** Dynamic web-based nomogram for predicting HVLNM in cN0 PTC patients.

The logistic prediction model was established as follows: logit(P) = -3.273 - 0.042 ×age + 2.529 × (if nodule max-diameter ≥10mm)+ 3.496 × (if ACR scores ≥11 points)+ 1.405 ×(if nodule with heterogeneous enhancement). To enhance clinical utility and improve interpretability, relevant nomogram was constructed using R statistical software with the “rms” package and incorporate an individual case into the nomogram(**Figure 2**). Finally, we applied and registered a web-based nomogram: https://qiannomogram.shinyapps.io/dynnomapp/ (**Figure 3**).

### 3.4 Evaluation of the model

**Figure 4:**
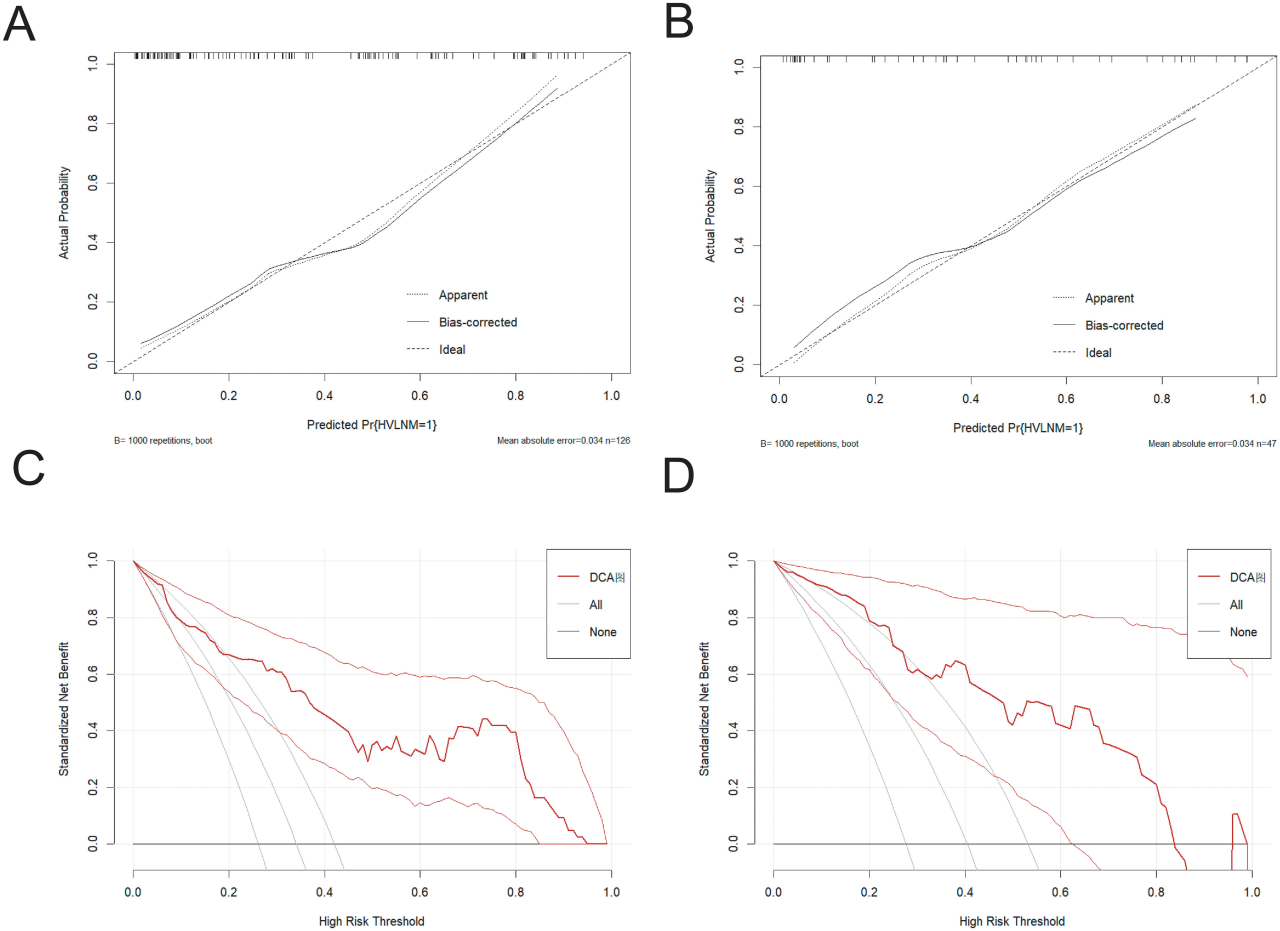
**(A, B)** The calibration curve in the training set and the external test set. **(C, D)** The DCA curve in the training set and the external test set.

The nomogram demonstrated an area under the curve (AUC) of 0.860 (95% CI: 0.792–0.928, P < 0.001), indicating excellent predictive accuracy. Model calibration was assessed using the Bootstrap method with 1000 resamples, showing close agreement between predicted and observed outcomes in training and external test sets (**Figures 4A, B**), thereby confirming model’s robust calibration performance.

### 3.5 Clinical assessment and implementation

**Figure 5:**
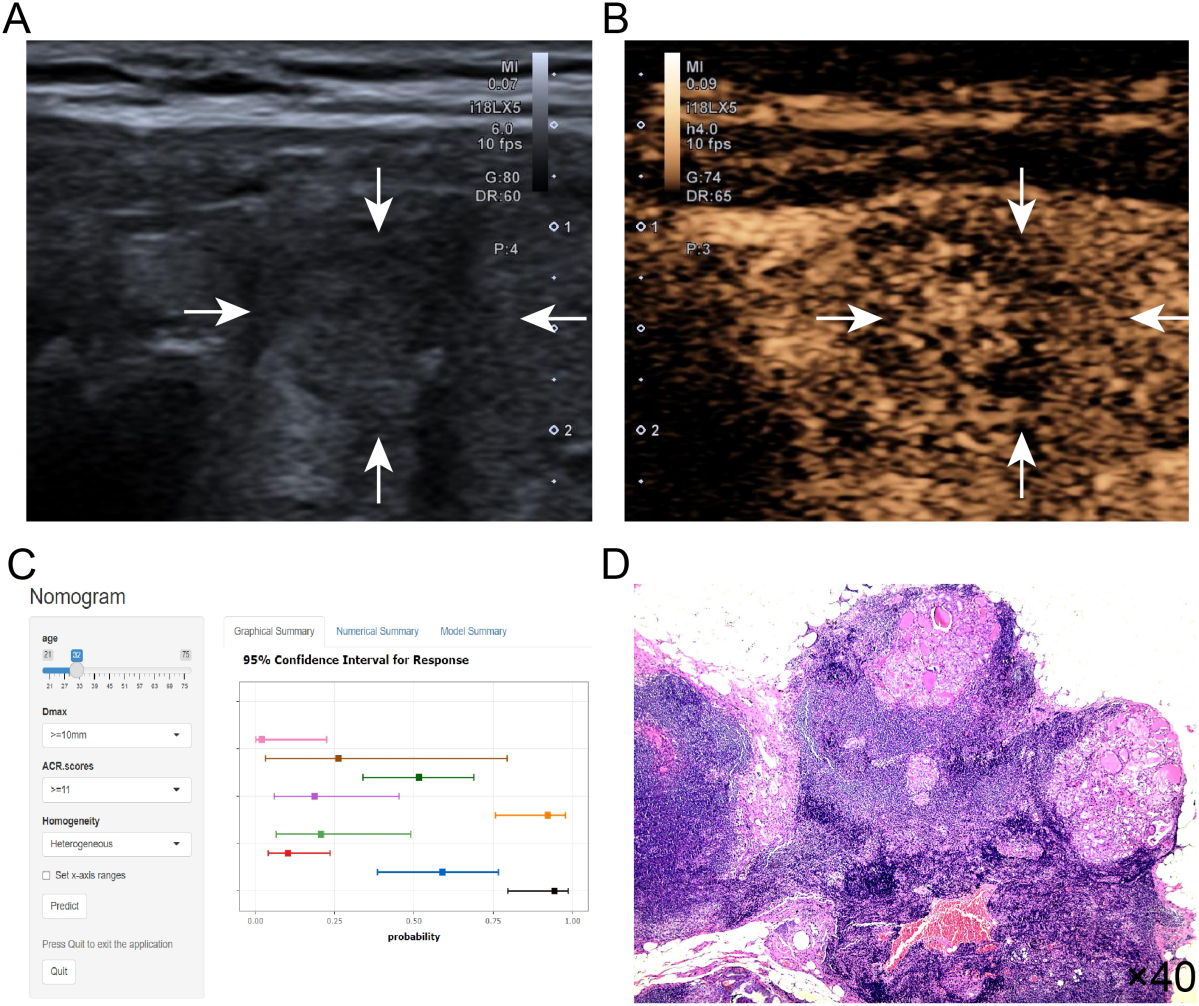
**(A–D)** Title: An example of dynamic web-based nomogram for predicting a 32-year-old male PTC patient with HVLNM. **(A)** Conventional ultrasound demonstrated a 13mm hypoechoic nodule (2 points) in the right thyroid lobe with the following ACR TI-RADS features: solid composition (2 points), taller-than-wide shape (3 points), irregular/lobulated margins (2 points) and microcalcifications (3 points), yielding ACR scores of ≥11 points. **(B)** CEUS showed heterogeneous contrast enhancement of this thyroid nodule. **(C)** Dynamic web-based nomogram predicted that the risk of HVLNM in this patient was 0.889. **(D)** Pathology showed HVLNM in this PTC patient.

The Decision curve analysis (DCA) curves showed a threshold probability from 10% to 93% in the training set, suggesting our nomogram offered greater clinically net-benefit compared to the extreme curve and individual ultrasonographic parameters, indicating its enhanced clinical utility for predicting HVLNM in PTC patients (**Figures 4C, D**). The clinical applicability of our model was further illustrated through a representative case study, showcasing the dynamic prediction of HVLNM risk in PTC patients using the web-based nomogram platform (**Figure 5**).

### 3.6 Internal and external validation

A ten-fold cross-validation approach was implemented, wherein the dataset was partitioned into 10 subsets, with 9 subsets utilized for model training and the remaining 1 subset for validation in each iteration. This process was repeated for 10 cycles to ensure robust evaluation. The model demonstrated an overall accuracy of 0.764 and a kappa coefficient of 0.468. The accuracy exceeding 0.7 indicates satisfactory predictive performance of predictive model, and the kappa value of 0.468 suggests moderate reliability and consistency. The ROC value of the external test was 0.885(95% CI:0.792-0.978), revealing the robust discriminative power.

## 4 Discussion

Accurate prediction of the burden of cervical lymph node metastasis significantly influences the prognosis in PTC (20). As reported by the Randolph et al (9), the prognostic stratification of LNM in PTC can be established based on metastatic lymph node quantity and other clinicopathological factors, with >5 pathological LNMs correlating with increased recurrence risk. Accurate preoperative identification of high-volume lymph node metastasis (HVLNM) enables appropriate radical lymph node dissection, thereby preventing secondary surgical interventions and reducing complication risks, including neck hematoma, surgical site infection, recurrent laryngeal nerve injury, and permanent hypoparathyroidism. Although numerous investigations have explored the association between sonographic characteristics and LNM in PTC, limited research has specifically addressed HVLNM prediction before the surgery. To deal with these clinical needs, we developed and validated a comprehensive nomogram incorporating ACR TI-RADS scores, multimodal ultrasonographic features, and clinicopathological parameters for evaluating HVLNM probability in PTC patients before the surgery.

Age is the first odds ratio (OR) value of the risk factors to enter the logistic regression model. Clinical studies have revealed that younger PTC patients have higher risk of cervical lymph node metastases than older patients, and the incidence of HVLNM is also relatively higher in younger patients (21). This trend has also been verified in multiple large cohort studies, may be attributed to the distinct biological characteristics of tumor cells in younger patients, including higher proliferation rates, enhanced invasiveness, and stronger metastatic capability. Additionally, young patients may experience atypical or overlooked early symptoms, leading to tumor progression and extensive lymph node metastases.

Max nodule diameter (Dmax) is a significant prognostic factor in papillary thyroid carcinoma (PTC) with relevant clinical data indicated that tumor ≥10mm in diameter exhibit greater aggressiveness and a significantly higher incidence of lymph node metastasis (LNM) (22). Liu et al. (23) developed a clinical nomogram incorporating independent risk factors with maximal nodule diameter and specific ultrasonographic characteristics. These collective results suggest that increased nodule diameter correlates with greater tumor aggressiveness and higher propensity for metastatic spread to regional lymph nodes. Our findings further support this observation: in the training cohort, the rate of high-volume lymph node metastasis(HVLNM) was 13.2% in patients with tumors <10 mm, compared to 58.6% in those with tumors ≥10 mm. Multivariate regression analysis confirmed that a tumor diameter ≥10 mm is an independent risk factor for HVLNM (OR=12.544, 95% CI: 4.488-35.06, P<0.001), indicating a strong positive correlation between tumor size and HVLNM risk.

The Thyroid Imaging Reporting and Data System (TI-RADS), introduced by the American College of Radiology (ACR) in 2017 (18), is a standardized classification framework that assigns malignancy risk scores based on a comprehensive evaluation of conventional ultrasonographic features of thyroid nodules. This scoring system not only facilitates the categorization of nodules but also provides detailed quantitative risk stratification, thereby enhancing diagnostic accuracy for PTC (24). Emerging evidence suggests that the ACR scores may serve as an independent predictive factor for PTC characteristics. For example, Chen et al. (25) reported that higher ACR TI-RADS scores, categorized into ten levels (≤5 points, 6–10 points, and ≥11 points), are significantly related to central neck lymph node metastasis (CNLNM) in PTC patients, confirming the potential relevance and predictive value of the ACR scores in metastatic lymph nodes.

Zhao et al. (26) conducted a study on 367 thyroid nodules from 307 patients by using preoperative multimodal ultrasound examination, identifying heterogeneous enhancement as an independent risk factor for HVLNM and establishing a multimodal diagnostic model for HVLNM. Heterogeneous contrast enhancement in malignant nodules reflects irregular perfusion patterns (27), primarily attributed to abnormal neovascularization (characterized by higher peripheral micro-vessel density creating uneven contrast distribution with poorly defined margins) and mechanical compression from tumor expansion (which distorts surrounding vasculature, amplifying filling heterogeneity) (28). The heterogeneous filling of contrast agent holds dual significance in the diagnosis of PTC patients with HVLNM. On the one hand, it provides more detailed information on tumor vascular distribution (29), it may simultaneously obscure tumor boundaries and increase diagnostic uncertainty (30). In this study, heterogeneous contrast agent filling demonstrated a close intrinsic connection with the prognosis of PTC patients with HVLNM and provided reference information for clinical decision-making.

Xiao et al. (31) established a multimodal radiopathomics model integrating ultrasound and FNAC-derived radiomics/pathomics features with six machine learning algorithms, demonstrating excellent predictive performance for high-volume CNLNM in cN0 PTC. Lin et al. (32) identified age, nodule diameter, BRAFV600E mutation, and calcification as independent predictors of CNLNM through logistic regression analysis of 57 PTC cases, subsequently developing a preoperative nomogram. The former study finding differ from ours in that we did not consider extracting radiomics and pathomics features. The results of the latter study differ from ours, *as* our differential analysis demonstrated that BRAF V600E mutation status showed no statistically significant difference between the two cohorts (p=0.36). Potential explanations for this observation include sample size constraints, population heterogeneity, lifestyle differences, and variations in varied detection methods.

Several limitations should be acknowledged. First of all, as a retrospective case-control study, the outcomes may be subject to potential selection bias inherent to the study design. Secondly, we acknowledge technical constraints of single-timepoint CEUS evaluation imposed by contrast agent costs and patient compliance, and inherent diagnostic variability in qualitative CEUS assessment despite standardized interpretation protocols. Thirdly, the limited sample size may reduce statistical power, necessitating extended patient recruitment and validation in future cohorts. Finally, the predictive performance of the model could potentially be enhanced by integrating other imaging modalities, such as elastography ultrasound or superb microvascular imaging (SMI). Future work will involve a prospective case-control study with a larger, geographically diverse cohort. Meanwhile, standardized ultrasound protocols (including elastography and SMI) will be employed to evaluate the roles of nodule elasticity, vascular patterns, and microvascular structure.

In conclusion, high-volume lymph node metastasis (HVLNM) represents an adverse prognostic indicator in PTC, with surgical approach significantly influencing patient outcomes. Our study demonstrates that the prediction model serves as an effective visual tool for preoperative HVLNM assessment in clinically lymph node-negative PTC patients and assists clinicians potentially reducing reoperation rates through more accurate initial interventions.

## Data Availability

The raw data supporting the conclusions of this article will be made available by the authors, without undue reservation.
