# Supplementary material for: Multimodal ultrasonographic and clinicopathological model for predicting high-volume lymph node metastasis in cN0 papillary thyroid carcinoma
Source: Front Endocrinol (Lausanne). 2025 Aug 21;16:1613672. doi: 10.3389/fendo.2025.1613672 (PMC12408322; doi:10.3389/fendo.2025.1613672)
Supplement: Supplementary file 1 [file Table1.pdf]

Supplementary Table 1 Baseline characteristics of the training cohort before and after propensity score matching

Variables	Unmatched cohort			After propensity score matching		
	HVLNM (n=52)	Non-HVLNM (n=147)	*P*	HVLNM (n=43)	Non-HVLNM (n=83)	*P*
BMI	20.1(18.4~23.1)	19.8(17.8~22.8)	0.311	20.4(18.3~23.2)	19.9(17.5~22.9)	0.396
Age	40(34~47.75)	48(39~57)	**0.001**	38(34~48)	47(38~57.25)	**0.01**
Gender			0.852			0.60
male	18()	53		16(37.2)	27(32.5)	
female	34	94		27(62.8)	56(67.5)	
FT3 (pmol/L)	5.1(4.54~5.89)	5.22(4.76~5.59)	0.614	5.06(4.48~5.93)	5.23(4.76~5.60)	0.78
FT4 (pmol/L)	17.5(16.1~19.4)	17.4(15.9~19.13)	0.757	17.4(16.1~19.41)	17.38(15.86~19.17)	0.62
TSH (uIU/ml)	1.72(1.31~2.53)	2.1(1.39~2.83)	0.054	1.73(1.08~2.62)	2.035(1.35~2.84)	0.17
Tg-Ab (IU/ml)	16.9(13.01~53.2)	19.14(15.5~77.8)	0.167	17.3(15.2~91.4)	18.0(14.0~42.8)	0.60
TPO-Ab (IU/ml)	17.3(4.01~49.73)	5.3(1.51~24.6)	0.590	2.61(1.44~8.15)	2.8(0.96~37.18)	0.62
Tg (ng/ml)	29.55(7.68~65.6)	15.5(5~28.8)	**0.012**	30.7(7.4~68.8)	14.1(6.3~27.75)	**0.008**
TR-Ab (U/L)	0.26(0.25~0.48)	0.33(0.25~0.55)	0.181	0.25(0.25~0.46)	0.335(0.25~0.53)	0.09
CT (pg/ml)	3.96(2.0~6.44)	3.25(2.0~7.27)	0.601	3.04(2~6.11)	3.46(2~8.89)	0.78
CEA			0.295			0.328
normal	47(90.4)	139(94.6)		39(90.7)	79(95.2)	
high	5(9.6)	8(5.4)		4(9.3)	4(4.8)	
BRAF gene			0.218			0.38

positive	48(92.3)	126(85.7)		40(93)	73(88)	
negative	4(7.7)	21(14.3)		3(7)	10(12)	
TI-RADS			**0.047**			0.343
4a	12(23.1)	53(36.1)		11(25.6)	32(38.6)	
4b	29(55.8)	80(54.4)		25(58.1)	42(50.6)	
4c	11(21.1)	14(9.5)		7(16.3)	9(10.8)	
Diffuse lesions			0.338			0.68
present	10(19.2)	38(25.9)		7(16.3)	16(19.3)	
absent	42(80.8)	109(74.1)		36(83.7)	67(80.7)	
